# Mental Health Morbidities and Time to Cancer Diagnosis Among Adults With Colon Cancer in England

**DOI:** 10.1001/jamanetworkopen.2022.38569

**Published:** 2022-10-31

**Authors:** Sara Benitez Majano, Georgios Lyratzopoulos, Niek J. de Wit, Becky White, Bernard Rachet, Charles Helsper, Juliet Usher-Smith, Cristina Renzi

**Affiliations:** 1 Inequalities in Cancer Outcomes Network Group, Department of Non-communicable Disease Epidemiology, London School of Hygiene and Tropical Medicine, London, United Kingdom; 2 Epidemiology of Cancer Healthcare and Outcomes Research Group, Department of Behavioural Science and Health, Institute of Epidemiology and Health Care, University College London, London, United Kingdom; 3 Julius Center for Health Sciences and Primary Care, University Medical Center, Utrecht University, Utrecht, the Netherlands; 4 Primary Care Unit, Department of Public Health and Primary Care, University of Cambridge, Cambridge, United Kingdom; 5 Faculty of Medicine, University Vita-Salute San Raffaele, Milan, Italy

## Abstract

**Importance:**

Mental health morbidity (MHM) in patients presenting with possible cancer symptoms may be associated with prediagnostic care and time to cancer diagnosis.

**Objective:**

To compare the length of intervals to cancer diagnosis by preexisting MHM status in patients who presented with symptoms of as-yet–undiagnosed colon cancer and evaluate their risk of emergency cancer diagnosis.

**Design, Setting, and Participants:**

This cohort study was conducted using linked primary care data obtained from the population-based Clinical Practice Research Datalink, which includes primary care practices in England, linked to cancer registry and hospital data. Included participants were 3766 patients diagnosed with colon cancer between 2011 and 2015 presenting with cancer-relevant symptoms up to 24 months before their diagnosis. Data analysis was performed in January 2021 to April 2022.

**Exposures:**

Mental health conditions recorded in primary care before cancer diagnosis, including anxiety, depression, schizophrenia, bipolar disorder, alcohol addiction, anorexia, and bulimia.

**Main Outcomes and Measures:**

Fast-track (also termed 2–week wait) specialist referral for investigations, time to colonoscopy and cancer diagnosis, and risk of emergency cancer diagnosis.

**Results:**

Among 3766 patients with colon cancer (median [IQR] age, 75 [65-82] years; 1911 [50.7%] women ), 623 patients [16.5%] had preexisting MHM recorded in primary care the year before cancer diagnosis, including 562 patients (14.9%) with preexisting anxiety or depression (accounting for 90.2% of patients with preexisting MHM) and 61 patients (1.6%) with other MHM; 3143 patients (83.5%) did not have MHM. Patients with MHM had records of red-flag symptoms or signs (ie, rectal bleeding, change in bowel habit, or anemia) in the 24 months before cancer diagnosis in a smaller proportion compared with patients without MHM (308 patients [49.4%] vs 1807 patients [57.5%]; *P* < .001). Even when red-flag symptoms were recorded, patients with MHM had lower odds of fast-track specialist referral (adjusted odds ratio [OR] = 0.72; 95% CI, 0.55-0.94; *P* = .01). Among 2115 patients with red-flag symptoms or signs, 308 patients with MHM experienced a more than 2-fold longer median (IQR) time to cancer diagnosis (326 [75-552] days vs 133 [47-422] days) and higher odds of emergency diagnosis (90 patients [29.2%] vs 327 patients [18.1%]; adjusted OR = 1.63; 95% CI, 1.23-2.24; *P* < .001) compared with 1807 patients without MHM.

**Conclusions and Relevance:**

This study found that patients with MHM experienced large and prognostically consequential disparities in diagnostic care before a colon cancer diagnosis. These findings suggest that appropriate pathways and follow-up strategies after symptomatic presentation are needed for earlier cancer diagnoses and improved health outcomes in this large patient group.

## Key Points

**Question:**

Do patients with preexisting anxiety, depression, or other mental health conditions experience disparities in care before the diagnosis of colorectal cancer?

**Findings:**

In this cohort study of 3766 patients diagnosed with colon cancer, those with mental health conditions and as-yet–undiagnosed cancer were 28% less likely to be promptly investigated with colonoscopy despite red-flag symptoms. Anxiety and depression were associated with more than 2-fold longer diagnostic intervals and 63% higher odds of emergency diagnosis, independently of physical comorbidity, age, and socioeconomic deprivation.

**Meaning:**

These findings suggest that patients with mental health morbidity experience prognostically consequential disparities in the diagnosis of cancer, highlighting the urgent need of improved diagnostic and follow-up strategies for this large patient group.

## Teaser

This cohort study investigates the association of preexisting mental health morbidities with time to diagnosis among adults with colon cancer in England.

## Introduction

Patients with preexisting mental health morbidity (MHM) are more likely to die prematurely from cancer.^1,2,3^ Mental health conditions are among the most frequent morbidities in Western populations, with 1 in 4 adults self-reporting mental health problems^4^ and 1 in 8 adults (13%) presenting symptoms of anxiety or depression to primary care.^5^ MHM has been associated with advanced-stage cancer.^6^ However, little is known about how MHM may be associated with the diagnostic process, from symptomatic presentation to timely investigations and diagnostic intervals.^7,8,9,10^ Diagnostic delays may occur when symptoms (eg, fatigue or abdominal pain, both possible colorectal cancer symptoms) are attributed to the preexisting MHM, which offers an alternative explanation.^8^ MHM may also be associated with delayed invasive investigations, such as colonoscopy, due to patient fear or anxiety^11^ or competing priorities among patients and clinicians when managing complex clinical needs.

Diagnosing cancer early, before it becomes a medical emergency, is paramount for improving survival. A 2022 population-based study^12^ on colon cancer cohorts in 14 jurisdictions in Australia, Canada, New Zealand, Norway, Denmark, and the UK found that colon cancer diagnoses after emergency presentations occurred in between 23% and 36% of patients and were associated with 3-fold greater odds of 1-year mortality compared with nonemergency routes. A 4-month diagnostic delay of colorectal cancer may be associated with a 20% reduction of 10-year survival.^13,14^ The COVID-19 pandemic was associated with delays in cancer investigations,^13,15^ with an estimated 17% increase in colorectal cancer deaths.^15^ It is estimated that after the pandemic, nearly 10 million people in England will have additional mental health needs,^16^ meaning patients with MHM may experience doubly negative outcomes.

This study focused on patients presenting with possible cancer symptoms to primary care given that approximately 85% of colon cancers are diagnosed after symptomatic presentations rather than screening.^17,18^ We aimed to examine variations in symptomatic presentations and subsequent diagnostic care by preexisting MHM status among patients with as-yet–undiagnosed colon cancer to characterize their odds of prolonged time to cancer diagnosis and emergency diagnosis.

## Methods

This cohort study was approved by the UK Medicines and Healthcare Products Regulatory Agency Independent Scientific Advisory Committee for database research, which enables regulated access for research of anonymous data from the Clinical Practice Research Datalink (CPRD), for which there is no requirement for informed consent. The study was performed in accordance with the Declaration of Helsinki and followed the Strengthening the Reporting of Observational Studies in Epidemiology (STROBE) reporting guideline.

### Study Population and Data Sources

**Figure 1.**
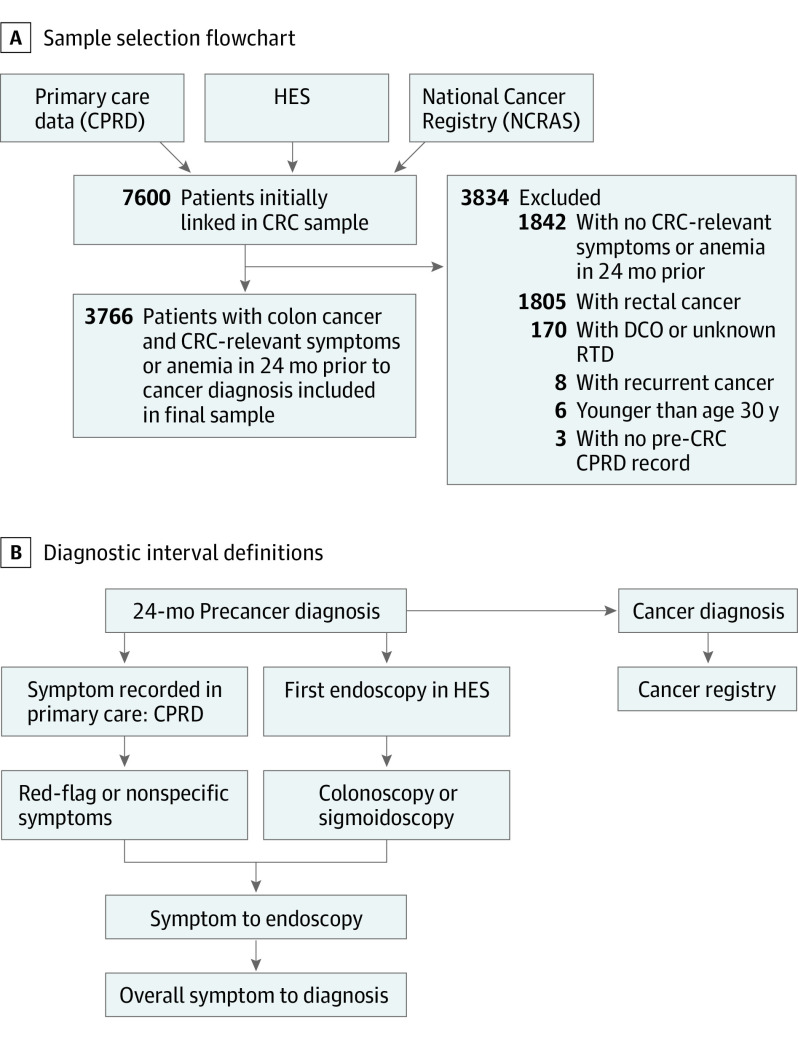
Study Sample, Data Sources, and Diagnostic Interval Definition Colorectal cancer (CRC)–relevant symptoms included red-flag symptoms and signs (ie, rectal bleeding, change in bowel habit, and anemia) and nonspecific symptoms (eg, abdominal pain, constipation, diarrhea, weight loss, fatigue). CPRD indicates Clinical Practice Research Datalink; DCO, death certificate only; HES, Hospital Episode Statistics; NCRAS, National Cancer Registration and Analysis Service; RTD, route to diagnosis.

We performed a cohort study of patients aged 30 to 99 years diagnosed with colon cancer (*International Statistical Classification of Diseases and Related Health Problems, Tenth Revision* [*ICD-10*] codes C18.1-C18.9) between 2011 and 2015 using National Cancer Registry records linked to primary care data (CPRD) and Hospital Episode Statistics (HES) outpatient and admitted patient data. We included 3766 patients with at least 1 colon cancer–relevant symptom recorded in primary care during the 24 months before cancer diagnosis (symptom list in eTable 1 in the Supplement). Nonsymptomatic screening–detected cancers or emergency diagnoses without prior symptomatic primary care presentation were excluded. We also excluded rectal cancers and focused specifically on colon cancer given that diagnostic delays and emergency and advance-stage diagnoses occur more frequently in colon cancer (Figure 1).^19^

CPRD includes more than 670 UK general practices, is representative of the general population, and provides prospectively collected patient-level information on signs and symptoms, diagnoses, and tests.^20^ Cancer site and diagnosis date were obtained from the National Cancer Registry. Data linkage was performed by the trusted third party National Health Service Digital.^20^ Data analysis was performed in January 2021 to April 2022.

### Study Variables

The main explanatory variables were MHM recorded in primary care before cancer diagnosis. Validated code lists and definitions were obtained from the Cambridge Multimorbidity Score.^5^ Specifically, for anxiety or depression, read codes and 4 or more anxiolytic, hypnotic, or antidepressant prescriptions in last 12 months were considered.^5^ A combination of anxiety and depression is common, and treatments can overlap. We therefore examined anxiety, depression, or both grouped as 1 condition, in line with the Cambridge multimorbidity definition.^5^ For schizophrenia or bipolar disorder, read codes or lithium prescription ever recorded were considered; alcohol problems, anorexia, and bulimia were defined based on read codes ever recorded.^5^

Further explanatory variables were red–flag colon cancer symptoms or signs, including rectal bleeding, change in bowel habit, or laboratory-confirmed anemia recorded in primary care before cancer; we also examined non–red-flag symptoms (eg, abdominal pain, constipation, diarrhea, weight loss, and fatigue).^21^ Change in bowel habit, rectal bleeding, and anemia are considered red-flag symptoms or signs warranting fast-track referral for investigations according to current guidelines.^21^ Relevant symptoms and signs (and related read codes or medcodes) were defined based on the literature and clinical expert revisions.^7,19,21^ Similar to previous work,^19^ we classified symptoms as new onset if recorded for the first time during the 24 months before cancer diagnosis without records of the same symptom in the previous 3 to 5 years or as chronic symptoms if recorded during the 24 months and 3 to 5 years before cancer. Anemia was defined based on hemoglobin tests below sex-specific thresholds provided by CPRD.

Given that physical comorbidity may be associated with timeliness of diagnostic endoscopy,^19^ we calculated the Charlson Comorbidity Index score using a validated algorithm,^22^ identifying morbidity *ICD-10* codes within HES inpatient and outpatient records. Additionally, we identified benign gastrointestinal (GI) conditions (ie, irritable bowel syndrome [IBS], diverticular disease [DD], and peptic ulcer) recorded in primary care before cancer given that these may be associated with MHM^16^ and timely cancer diagnosis.^8^ We defined these conditions following Cambridge definitions;^5^ for example, for IBS we considered read codes ever before cancer or 4 or more antispasmodic prescriptions in the last 12 months. Additionally, we included the number of primary care consultations for any reason during the year before cancer and sociodemographic characteristics (ie, sex, age, and socioeconomic deprivation using the Index of Multiple Deprivation 2015).

#### Primary Outcome Measures

The main outcomes were fast-track (also known as 2–week wait) referral for cancer investigations and emergency cancer diagnosis, defined according to the validated Routes to Diagnosis algorithm (eAppendix in the Supplement).^17,23^ Fast-track general practitioner (GP) referrals included patients referred urgently for suspected cancer to see a specialist within 2 weeks (introduced in England in 2000). Emergency cancer diagnoses included diagnoses after presentation to accident and emergency units or through GP emergency referral or emergency pathways for inpatients and outpatients.^17,23^ Additional outcomes included the overall symptomatic consultation to diagnosis interval, calculated similarly to what was done in previous work^19^ for all patients with new-onset symptoms as the time in days from first new-onset relevant symptom recorded in CPRD during the 24 months before cancer diagnosis (index symptom) to cancer diagnosis (Figure 1). Secondary outcomes included use of colonoscopy or flexible sigmoidoscopy and interval from symptomatic consultation to endoscopy (days from index symptom to endoscopy). Endoscopy information was extracted from HES records in the 24 months before cancer and after a cancer-relevant symptom in primary care using OPCS 4.5 Standard Classification for National Health Services procedures codes (code list available upon request). A 2019 study^24^ found 96% accuracy for HES data on investigations. Key variables and hypothesized associations among MHM, symptomatic presentations, and diagnostic routes are illustrated in the eFigure in the Supplement.

### Statistical Analysis

We described sociodemographic characteristics, symptoms, physical comorbidity burden, number of GP consultations for any reason before cancer, endoscopy use, and diagnostic route by MHM status. The frequency distribution by MHM was compared using χ^2^ tests. We used multivariable logistic regression to assess associations between MHM and outcomes of interest (separate models for each outcome: fast-track referral, emergency cancer diagnosis, and first symptomatic consultation to diagnosis interval). This analysis accounted for variables thought a priori to be potentially associated based on the literature and clinical reasoning: symptoms, comorbidity burden, benign GI conditions, number of consultations, and sociodemographic characteristics. Given that general practices may vary in the use of endoscopy,^25^ analyses accounted for patient clustering by practice and estimated robust standard errors.

We used quantile multivariable regression to examine variations in diagnostic intervals by MHM, accounting for symptoms, comorbidities, number of consultations, age, sex, and socioeconomic deprivation. This allowed us to compare diagnostic intervals by MHM and other patient characteristics by centile (50%, 25%, and 75%) of diagnostic intervals. Similar to previous research, we focused on the median (fiftieth centile) and seventy-fifth centile.^19^

According to UK guidelines, fast-track referrals are warranted for patients with red-flag symptoms or signs.^21^ Thus, subanalyses focused on patients with red-flag symptoms or signs and examined the likelihood of fast-track referral by MHM status, accounting for patient characteristics. Because fast-track referral is recommended for patients with colon cancer red-flag symptoms or signs aged 60 years or older,^21^ we additionally examined this patient group. We used Stata statistical software version 16 (StataCorp) for statistical analyses. We used 2-sided tests and considered *P* < .05 as statistically significant.

## Results

Among 3766 patients with colon cancer (median [IQR] age, 75 [65-82] years; 1911 [50.7%] women), 623 patients (16.5%) had preexisting MHM recorded in primary care, including 562 patients (14.9%) with anxiety or depression documented in the year before cancer diagnosis (accounting for 90.2% of patients with preexisting MHM) and 61 patients (1.6%) with other MHM; 3143 patients (83.5%) did not have preexisting MHM. Overall, 860 patients (22.8%) had 1 or more new–onset red-flag symptom (ie, rectal bleeding or change in bowel habit), 1255 patients (33.3%) had lab-test anemia, and 1220 patients (32.4%) had only non–red-flag symptoms (eg, abdominal pain or fatigue) recorded before cancer (Table 1). Patients with MHM had records of red-flag symptoms or signs (ie, rectal bleeding, change in bowel habit, or anemia) in the 24 months before cancer diagnosis in a smaller proportion compared with patients without MHM (308 patients [49.4%] vs 1807 patients [57.5%]; *P* < .001).

**Table 1.** Patient Characteristics and Route to Cancer Diagnosis

Characteristic	Patients, No. (%)			χ^2^ *P* value
	Total (N = 3766)	No MHM (n = 3143)	MHM (n = 623)	
Age group, y				
<45	85 (2.3)	72 (2.3)	13 (2.1)	.001
45-54	254 (6.7)	210 (6.7)	44 (7.1)	
55-64	539 (14.3)	474 (15.1)	65 (10.4)	
65-74	941 (25.0)	795 (25.3)	146 (23.4)	
75-84	1317 (35.0)	1099 (35.0)	218 (35.0)	
≥85	630 (16.7)	493 (15.7)	137 (22.0)	
Sex				
Men	1855 (49.3)	1619 (51.5)	236 (37.9)	<.001
Women	1911 (50.7)	1524 (48.5)	387 (62.1)	
Socioeconomic deprivation quintile				
1 (least deprived)	946 (25.1)	778 (24.8)	168 (27.0)	<.001
2	871 (23.1)	759 (24.1)	112 (18.0)	
3	805 (21.4)	685 (21.8)	120 (19.3)	
4	656 (17.4)	541 (17.2)	115 (18.5)	
5 (most deprived)	488 (13.0)	380 (12.1)	108 (17.3)	
CCI score				
0	1957 (52.0)	1712 (54.5)	245 (39.3)	<.001
1	875 (23.2)	698 (22.2)	177 (28.4)	
2	418 (11.1)	335 (10.7)	83 (13.3)	
≥3	516 (13.7)	398 (12.7)	118 (18.9)	
IBS or DD				
No	3432 (91.1)	2881 (91.7)	551 (88.4)	.01
Yes	334 (8.9)	262 (8.3)	72 (11.6)	
Visits to GP in 1-12 mo before cancer diagnosis, No.				
0	89 (2.4)	86 (2.7)	3 (0.5)	<.001
1-4	328 (8.7)	310 (9.9)	18 (2.9)	
5-9	663 (17.6)	608 (19.3)	55 (8.8)	
≥10	2686 (71.3)	2139 (68.1)	547 (87.8)	
Symptom in 24 mo before cancer diagnosis				
Rectal bleeding or CIBH	860 (22.8)	741 (23.6)	119 (19.1)	<.001
Anemia (as the only red flag)	1255 (33.3)	1066 (33.9)	189 (30.3)	
Non–red-flag symptoms only	1220 (32.4)	1006 (32.0)	214 (34.3)	
Chronic symptoms only	431 (11.4)	330 (10.5)	101 (16.2)	
Bowel endoscopy in 24 mo before cancer diagnosis				
No	1266 (33.6)	1026 (32.6)	240 (38.5)	.005
Yes	2500 (66.4)	2117 (67.4)	383 (61.5)	
Route to diagnosis				
Emergency presentation	1090 (28.9)	859 (27.3)	231 (37.1)	<.001
Fast track	1176 (31.2)	1031 (32.8)	145 (23.3)	
GP referral	922 (24.5)	751 (23.9)	171 (27.4)	
Screening	156 (4.1)	148 (4.7)	8 (1.3)	
Inpatient elective	119 (3.2)	102 (3.2)	17 (2.7)	
Other outpatient	303 (8.0)	252 (8.0)	51 (8.2)	

Abbreviations: CCI, Charlson Comorbidity Index; CIBH, change in bowel habit; DD, diverticular disease; GP, general practitioner; IBS, irritable bowel syndrome; MHM, Mental Health Morbidity.

### Patient Characteristics and Route to Cancer Diagnosis by MHM Status

Patients with MHM were more frequently women, older, and more socially deprived than those without MHM. Additionally, patients with MHM less frequently had records of new–onset red-flag symptoms or signs (ie, rectal bleeding, change in bowel habit, or anemia). They had a higher burden of physical comorbidities, benign GI diagnosis (ie, IBS and DD) records and more primary care consultations; in contrast, they less frequently had records of endoscopy (Table 1). Emergency presentation was the most frequent diagnostic route for patients with MHM.

Findings were similar in the subgroup of 2115 patients with new–onset red-flag symptoms or signs (ie, rectal bleeding, change in bowel habit, or anemia). Among these patients, the proportion of 308 patients with MHM who had an emergency cancer diagnosis was significantly higher (90 patients [29.2%] vs 327 patients [18.1%]) and the proportion with a fast-track referral was significantly lower (94 patients [30.5%] vs 765 patients [42.3%]) compared with 1807 patients without MHM (*P* for route to diagnosis < .001) (eTable 2 in the Supplement).

### Diagnostic Intervals

**Figure 2.**
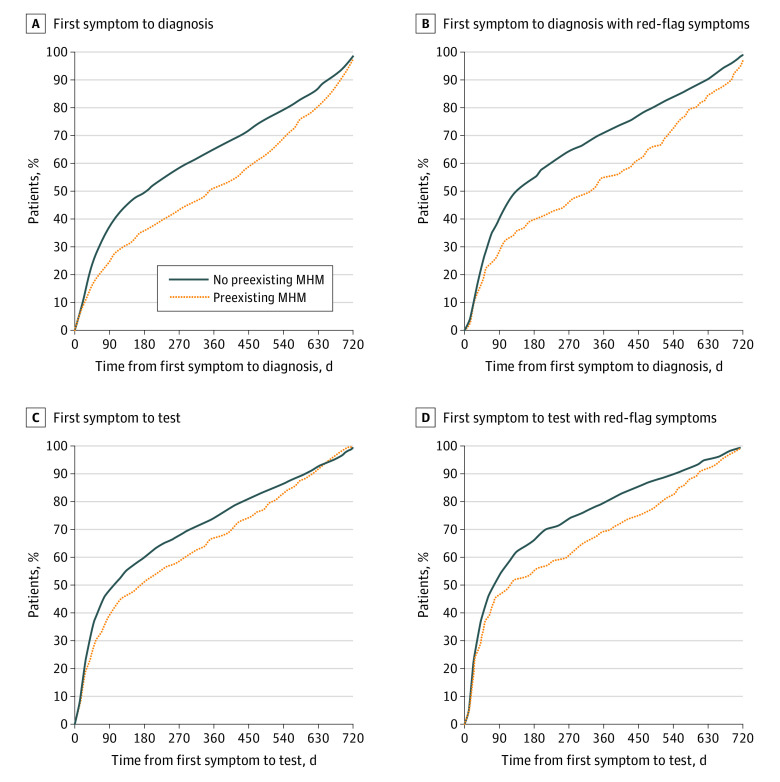
Diagnostic Intervals by Mental Health Morbidity (MHM) Status The symptomatic consultation to cancer diagnosis interval is presented for A, 3766 patients with symptomatic colon cancer and B, the subgroup of 2115 patients with new–onset red-flag symptoms or signs. The symptomatic consultation to endoscopy interval is presented for C, 2500 patients with symptomatic colon cancer who had an endoscopy and D, the subgroup of 1730 patients with red-flag symptoms or signs.

Among patients with cancer, those with vs without MHM had longer diagnostic intervals before cancer diagnosis; the median (IQR) symptomatic consultation to diagnosis interval was 350 [92-579] days vs 186 (50-484) days (Figure 2A), and median (IQR) symptomatic consultation to investigation interval was 173 (43-461) days vs 100 (30-370) days (Figure 2C). Similarly, among patients with new–onset red-flag symptoms, those with MHM had longer intervals; the median (IQR) symptomatic consultation to diagnosis interval was 326 (75-552) days vs 133 (47-422) days (Figure 2B), and the median (IQR) symptomatic consultation to investigation interval was 118 (29-453) days vs 72 (26-287) days (Figure 2D). Details on the number of patients at risk per period are listed in eTable 3 in the Supplement). Among examined patient subgroups, only those with a Charlson Comorbidity Index score of 3 or higher had a similarly long median (IQR) interval (335 [110-571] days) (Table 2). In quantile regression, after accounting for covariables, we also found longer diagnostic intervals for individuals with MHM (fiftieth centile: adjusted interval, 224.4; 95% CI, 159.1-289.8; *P* = .003; seventy-fifth centile: adjusted interval, 466.5; 95% CI, 413.4-519.6; *P* < .001) (Table 2).

**Table 2.** Interval From First Consultation With Red-Flag Symptom or Sign to Cancer Diagnosis (n = 2115)

Patient group	Interval, d			Multivariable quantile regression			
	Median (50th centile)	IQR		50th centile		75th centile	
		25th Centile	75th Centile	Adjusted interval (95% CI)	*P* value	Adjusted interval (95% CI)	*P* value
MHM							
No	133	47	422	Reference^a^	NA	Reference^a^	NA
Yes	326	75	552	224.4 (159.1-289.8)	.003	466.5 (413.4-519.6)	<.001
Age group, y							
<45	196	56	454	230.6 (157.0-304.1)	.005	468.5 (319.2-617.8)	.18
45-54	110	37	356	126.7 (94.1-159.4)	.97	351.5 (282.7-420.3)	.69
55-64	100	39	342	Reference^a^	NA	Reference^a^	NA
65-74	111	44	441	112.9 (90.0-135.7)	.26	357.0 (297.8-416.2)	.78
75-84	186	56	456	119.6 (96.2-143.0)	.59	385.0 (326.8-443.2)	.51
≥85	258	86	519	171.4 (129.8-213.0)	.03	436.0 (368.4-503.6)	.04
Sex							
Men	134	46	433	Reference^a^	NA	Reference^a^	NA
Women	165	53	470	126.7 (107.4-146.1)	.94	361.5 (325.0-398.0)	.83
Socioeconomic deprivation quintile							
1 (least deprived)	136	44	414	Reference^a^	NA	Reference^a^	NA
2	148	51	464	139.9 (113.4-166.3)	.30	418.5 (357.1-479.9)	.09
3	131	45	441	137.3 (109.6-165.0)	.42	416.5 (356.6-476.4)	.10
4	138	53	436	136.0 (108.4-163.6)	.48	364.5 (315.2-413.8)	.97
5 (most deprived)	285	61	532	204.1 (151.0-257.3)	.004	443.0 (384.1-501.9)	.01
CCI score							
0	100	39	344	Reference^a^	NA	Reference^a^	NA
1	215	51	491	206.9 (160.3-253.4)	.001	440.5 (382.6-498.4)	.01
2	237	90	512	212.9 (164.4-261.3)	<.001	418.5 (352.9-484.1)	.11
≥3	335	110	571	275.3 (210.6-340.0)	<.001	459.0 (396.3-521.7)	.003
IBS or DD							
No	140	46	440	Reference^a^	NA	Reference^a^	NA
Yes	288	96	533	217.4 (154.4-280.5)	.005	469.5 (412.7-526.3)	<.001
Visits to GP 1-12 mo before cancer diagnosis, No.							
0	601	24	671	416.7 (297.9-535.5)	<.001	559.0 (493.6-624.4)	<.001
1-4	35	21	119	25.1 (−0.2-50.5)	<.001	90.0 (20.9-159.1)	<.001
5-9	63	31	225	46.3 (23.0-69.5)	<.001	156.5 (101.0-212.0)	<.001
≥10	215	72	483	Reference^a^	NA	Reference^a^	NA
Type of symptom 24 mo before cancer diagnosis							
Rectal bleeding or CIBH	134	42	464	Reference^a^	NA	Reference^a^	NA
Anemia (as only red flag)	167	54	442	123.4 (105.7-141.2)	.78	329.5 (293.5-365.5)	.05

Abbreviations: CCI, Charlson Comorbidity Index; CIBH, change in bowel habit; DD, diverticular disease; IBS, irritable bowel syndrome; MHM, mental health morbidity.

^a^ The reference group was men aged 55 to 64 years in the least socioeconomically deprived group with no comorbidities or MHM who had a CIBH or rectal bleeding. The adjusted interval for this group was 126.0 (95% CI, 94.5-157.5) for the fiftieth centile and 365.5 (95% CI, 288.6-442.4) for the seventy-fifth centile.

### Multivariable Analysis of Diagnostic Routes by MHM

Among patients with red-flag symptoms, preexisting MHM was independently associated with lower odds of fast-track cancer investigations compared with no MHM (adjusted odds ratio [OR] = 0.72; 95% CI, 0.55-0.94; *P* = .01) (Table 3). Younger age group (eg, ages <45 y vs 55-64 years: adjusted OR = 0.37; 95% CI, 0.17-0.84; *P* = .02), socioeconomic deprivation (eg, most vs least deprived: adjusted OR = 0.07; 95% CI, 0.48-0.95; *P* = .02), higher comorbidity burden (eg, ≥ 3 vs 0 comorbidities: adjusted OR = 0.46; 95% CI, 0.32-0.65; *P* < .001), IBS or DD diagnosis (adjusted OR = 0.72; 95% CI, 0.38-0.77; *P* = .001), and recorded anemia vs rectal bleeding or change in bowel habit (adjusted OR = 0.80; 95% CI, 0.65-0.97; *P* = .03) were also associated with decreased odds of fast-track investigations.

**Table 3.** Likelihood of Fast-Track Referral Among Patients With Red-Flag Symptoms or Signs (n = 2115)

Patient group	Fast-track referral, No. (%)	OR (95% CI)^a^		*P* value
		Unadjusted	Adjusted^b^	
MHM				
No	765 (42.3)	1 [Reference]	1 [Reference]	NA
Yes	94 (30.5)	0.60 (0.46-0.78)	0.72 (0.55-0.94)	.02
Age group, y				
<45	9 (24.3)	0.40 (0.19-0.84)	0.37 (0.17-0.84)	.02
45-54	62 (46.6)	1.08 (0.70-1.65)	1.13 (0.72-1.78)	.59
55-64	134 (44.8)	1 [Reference]	1 [Reference]	NA
65-74	217 (41.7)	0.88 (0.65-1.19)	1.10 (0.80-1.51)	.55
75-84	333 (42.2)	0.90 (0.67-1.21)	1.28 (0.95-1.74)	.11
≥85	104 (31.0)	0.55 (0.38-0.79)	0.83 (0.57-1.21)	.32
Sex				
Men	444 (41.8)	1 [Reference]	1 [Reference]	NA
Women	415 (39.4)	1.08 (0.70-1.65)	1.13 (0.72-1.78)	.91
Socioeconomic deprivation quintile				
1 (least deprived)	233 (42.4)	1 [Reference]	1 [Reference]	NA
2	196 (40.6)	0.93 (0.72-1.19)	0.88 (0.68-1.15)	.36
3	218 (47.1)	1.21 (0.95-1.55)	1.18 (0.92-1.51)	.20
4	132 (36.1)	0.77 (0.59-1.00)	0.76 (0.58-1.00)	.05
5 (most deprived)	80 (31.6)	0.63 (0.45-0.88)	0.67 (0.48-0.95)	.02
CCI score				
0	529 (45.8)	1 [Reference]	1 [Reference]	NA
1	188 (40.0)	0.79 (0.65-0.96)	0.92 (0.74-1.14)	.43
2	85 (34.4)	0.62 (0.46-0.84)	0.76 (0.56-1.04)	.09
≥3	57 (23.5)	0.36 (0.26-0.51)	0.46 (0.32-0.65)	<.001
IBS or DD				
No	807 (41.9)	1 [Reference]	1 [Reference]	NA
Yes	52 (27.5)	0.53 (0.37-0.74)	0.54 (0.38-0.77)	.001
Visits to GP in 1-12 mo before cancer diagnosis, No.				
0	9 (33.3)	0.88 (0.39-1.97)	0.96 (0.43-2.14)	.92
1-4	103 (57.5)	2.38 (1.72-3.31)	1.90 (1.35-2.67)	<.001
5-9	188 (51.2)	1.85 (1.48-2.31)	1.54 (1.21-1.94)	<.001
≥10	559 (36.3)	1 [Reference]	1 [Reference]	NA
Type of symptom in 24 mo before cancer diagnosis				
Rectal bleeding or CIBH	387 (45.0)	1 [Reference]	1 [Reference]	NA
Anemia (only red flag)	472 (37.6)	0.74 (0.61-0.89)	0.80 (0.65-0.97)	.02

Abbreviations: CCI, Charlson Comorbidity Index; CIBH, change in bowel habit; DD, diverticular disease; IBS, irritable bowel syndrome; MHM, mental health morbidity; NA, not applicable; OR, odds ratio.

^a^ Logistic regression ORs and 95% CIs are presented.

^b^ The adjusted model includes all variables listed in the table.

Among patients with red-flag symptoms or signs, those with MHM had higher odds of emergency diagnosis than those without MHM (adjusted OR = 1.63; 95% CI, 1.23-2.24; *P* < .001) (eTable 4 in the Supplement). Similarly, among 726 patients aged 60 years or older with red-flag symptoms or signs, MHM was associated with lower odds of fast-track investigations (76 patients [27.9%] vs 650 patients [42.4%; adjusted OR = 0.62; 95% CI, 0.46-0.84; *P* = .002) (eTable 4 in the Supplement).

Similarly, among all patients with colon cancer and any relevant symptom (not only red-flag symptoms), patients with MHM had higher odds of emergency diagnosis (adjusted OR = 1.38; 95% CI, 1.16-1.74; *P* = .001) (eTable 6 in the Supplement).

## Discussion

In this cohort study, having an MHM was associated with 2-fold longer intervals before a colon cancer diagnosis. For individuals with new–onset red-flag symptoms, it took a median of 326 days (almost 1 year) from first symptomatic consultation to the diagnosis of cancer if they had a preexisting MHM compared with 133 days (more than 4 months) for those without MHM. Having an MHM was associated with approximately 30% lower odds of prompt investigations after presentation with red-flag symptoms and 60% higher odds of cancer diagnosis through an emergency presentation, even after adjustment for other known risk factors. These findings suggest the possibility of missed opportunities for earlier diagnosis, especially but not exclusively among the large group of people with anxiety or depression.

### Interpretation in Context

Previous research found that MHM was associated with premature cancer death.^1,2,3,6^ However, little was known about how MHM may be associated with the diagnostic process. Findings from 1 UK study and 2 small Dutch studies^7,8,26,27^ suggested longer diagnostic intervals for patients with MHM. A 2022 Danish study^10^ found that MHM was associated with a higher probability of cancer diagnosis through unplanned admissions, but whether this could be explained by differences in symptomatic presentations or timeliness of investigations was not explored. We found that patients with MHM and as-yet–undiagnosed colon cancer less frequently had records of typical cancer symptoms; furthermore, even when they had red-flag symptoms, they experienced disparities in prediagnostic care regarding use of and time to colonoscopy and routes to cancer diagnosis.

Little is known on how GI symptoms vary by MHM status in the general population without cancer. A study^28^ on the association between MHM and IBS reported higher self-reported severity of GI symptoms in these patients, but the association between psychiatric and chronic GI conditions and symptoms is not well understood. MHM may be associated with reduced patient reporting or poorer interpretation and recording of potential cancer symptoms among doctors; symptoms, such as change in bowel habit, abdominal pain, or fatigue, may be considered outcomes associated with an underlying anxiety disorder^9^ or adverse effects associated with psychotropic medications and attributed to benign conditions.^8^ Such mechanisms may partially explain why individuals with MHM less frequently had records of red-flag symptoms (including change in bowel habit) and more frequently had IBS records. IBS may provide an alternative explanation for cancer-delaying referrals in some patients, with longer symptom to diagnosis intervals and a higher emergency diagnosis risk, as observed in our study and previous studies.^8,27^

Consistent with earlier research, we found that high physical comorbidity burden was associated with prolonged diagnostic intervals,^7,8^ lower odds of fast-track referrals^29^ and endoscopy,^3,19^ and higher odds of emergency presentation.^19^ While patients with MHM have been found to have higher comorbidity,^16^ our study findings suggest that MHM and physical comorbidities are independently associated with timely cancer investigations. Some responsible mechanisms may overlap, while others may be specific to MHM. Patients with MHM may be reluctant to undergo colonoscopy due to fear or anxiety,^11^ possibly associated with the lower odds of endoscopy observed in our study. Simultaneously, a high comorbidity burden may be associated with increased time before invasive investigations can be safely performed given that the risk of procedure-related complications needs to be appropriately managed.^30^ Our findings suggest that for patients with preexisting morbidities, including MHM and other comorbidities, competing demands may be associated with lower odds of timely investigations even in some patients with red-flag symptoms or signs, such as anemia or rectal bleeding.^8^

### Implications for Research and Practice

Our findings highlighted prolonged diagnostic intervals and possible missed opportunities for earlier diagnosis among patients with MHM presenting with red-flag cancer symptoms, which was associated with patient and health care factors. These may include difficulties in symptom appraisal and doctor-patient communication when making decisions on investigations, patient difficulties in adhering to recommendations (eg, bowel preparation for colonoscopy),^11^ complexities in the informed consent process when patients have severe MHM with cognitive and emotional issues,^31^ clinician cognitive bias, insufficient time and support during consultations, and limited availability of integrated pathways for patients with complex conditions.^6,8^ Some delays are difficult to avoid given that clinical presentation of MHM or medication-associated adverse effects may genuinely provide alternative explanations for cancer symptoms (eg, change in bowel habit); moreover, clinicians need to balance cancer risk with increased anxiety due to invasive procedures and cancer fear before referring patients with MHM for endoscopy.

To diagnose cancer earlier in the large number of patients with complex needs, improved support is necessary for patients and clinicians^1^ given that multiple factors and suboptimal follow-up strategies may be associated with prolonged diagnostic process.^27^ Information technology based on electronic health records may help GPs to identify patients with complex conditions and plan resources and time allocation, for example, by involving specialist nurses before or after visits who are dedicated to patients with MHM. Similar approaches have been suggested in the management of physical multimorbidity.^32^ Information technology could also facilitate the provision of safety nets. Greater integration between primary and secondary care and wider use of disease-management programs coordinated by specialist nurses could enhance health care access.^1^ Recently introduced Rapid Diagnostic Centres in the UK for patients with serious but nonspecific symptoms could also be beneficial in the case of diagnostic complexities due to MHM and multimorbidity.

Our study focused on patients diagnosed after symptomatic presentation given that they have worse outcomes than individuals detected via screening.^17^ Targeting patients who are symptomatic and facilitating screening are essential to diagnosing cancer earlier and improving outcomes.^33^ Future research based on interviews with patients and doctors examining the time between symptom onset and help-seeking (ie, the patient interval) and communication during clinical encounters may provide further insights into possible barriers to timely cancer investigations.

### Limitations

Although we used validated algorithms and population-based data, encompassing prospectively recorded primary care, hospital, and cancer registration data, this study has several limitations. MHM was defined based on validated algorithms, including primary care diagnoses and symptom codes and prescriptions, obtaining estimates similar to those of previous studies^5^; however, primary care records likely underestimate MHM because some individuals may not consult physicians or their conditions may not be recorded. Analyses by specific psychiatric diagnosis (eg, schizophrenia) and severity of MHM could not be performed due to small numbers and nonavailability of severity data. It is likely that delays in cancer diagnosis may be particularly pronounced in people with severe psychiatric conditions.^6,10^ We examined anxiety and depression grouped as 1 condition, in line with the Cambridge definition^5^; however, future studies could examine these conditions separately given that their associated outcomes in GI-symptom management and cancer diagnosis may differ. Relying on routinely collected coded electronic records (without access to free-text notes) may have underestimated the number of patients who were symptomatic.

Physical comorbidity burden was measured using the validated Charlson Comorbidity Index based on hospital-recorded morbidities^22^ without considering morbidities managed in primary care.^5^ By focusing on conditions severe enough to be recorded during hospital admissions before cancer, we accounted for morbidities likely associated with decision-making on invasive investigations, such as colonoscopy. Linked data were available to us up to 2015, and more recent data will be needed given persisting inequalities in diagnostic timeliness.

Given that our analyses included numerous explanatory and outcome variables, we limited the risk of overadjustment^6^ by using different models for each outcome (fast-track referrals, emergency presentation, and diagnostic intervals) and considering unadjusted and adjusted models. Similar to previous research,^19^ we focused on endoscopy use, which is well documented in HES.^24^ We did not examine the use of fecal calprotectin testing. A study^34^ reported on its limited value for the diagnostic workup of patients suspected of significant colorectal disease. However, future research could investigate this further. We were not able to distinguish between primary care visits with a GP and those with another health care professional (eg, a primary care nurse). However, if red-flag symptoms are recorded during any primary care visit, appropriate consultations and testing should follow promptly. Our study found that this was not the case for a large proportion of patients.

## Conclusions

This cohort study found that patients with MHM experienced large and prognostically consequential disparities in diagnostic care before a colon cancer diagnosis. Opportunities for earlier diagnosis may exist for a substantial subgroup of patients with colon cancer who also have anxiety, depression, or another MHM given that these patients were not investigated promptly for cancer despite presenting with red-flag symptoms. Our findings suggest that MHM may be independently associated with timely diagnosis of cancer, although physical comorbidity burden, older age, and social deprivation were also associated with lower odds of fast-track investigations. The prolonged time from presentation with red-flag cancer symptoms to cancer diagnosis suggests the need for improved diagnostic and follow-up strategies after symptomatic presentations to diagnose cancer earlier, especially but not exclusively among the large group of patients with mental health conditions.

## Notes

Supplement. **eTable 1.** Symptom Code List **eAppendix.** Routes to Diagnosis for Included Patients Who Were Symptomatic **eFigure.** Study Variables and Hypothesized Associations Between Mental Health Morbidity and Symptomatic Presentations and Route to Cancer Diagnosis **eTable 2.** Characteristics of 2115 Patients With Colon Cancer and Red-Flag Symptoms by Mental Health Morbidity (MHM) Status **eTable 3.** Patients at Risk for Diagnosis and Investigation by Mental Health Morbidity (MHM) per Time Point Before Cancer Diagnosis **eTable 4.** Likelihood of Being Diagnosed After Emergency Presentation (EP) in 2115 Patients With Red-Flag Symptoms **eTable 5.** Likelihood of Being Diagnosed After Expedited Referral in Patients Aged 60 y and Older With Red-Flag Symptoms **eTable 6.** Likelihood of Being Diagnosed After Emergency Presentation Among Patients With Colon Cancer Who Were Symptomatic
