# Supplementary material for: Mental Health Morbidities and Time to Cancer Diagnosis Among Adults With Colon Cancer in England
Source: JAMA Netw Open. 2022 Oct 31;5(10):e2238569. doi: 10.1001/jamanetworkopen.2022.38569 (PMC9623442; doi:10.1001/jamanetworkopen.2022.38569)
Supplement: Supplement. — eTable 1. Symptom Code List eAppendix. Routes to Diagnosis for Included Patients Who Were Symptomatic eFigure. Study Variables and Hypothesized Associations Between Mental Health Morbidity and Symptomatic Presentations and Route to Cancer Diagnosis eTable 2. Characteristics of 2115 Patients With Colon Cancer and Red-Flag Symptoms by Mental Health Morbidity (MHM) Status eTable 3. Patients at Risk for Diagnosis and Investigation by Mental Health Morbidity (MHM) per Time Point Before Cancer Diagnosis eTable 4. Likelihood of Being Diagnosed After Emergency Presentation (EP) in 2115 Patients With Red-Flag Symptoms eTable 5. Likelihood of Being Diagnosed After Expedited Referral in Patients Aged 60 y and Older With Red-Flag Symptoms eTable 6. Likelihood of Being Diagnosed After Emergency Presentation Among Patients With Colon Cancer Who Were Symptomatic [file jamanetwopen-e2238569-s001.pdf]

Supplemental Online Content

Benitez Majano S, Lyratzopoulos G, de Wit NJ, et al. Mental health morbidities and time to cancer diagnosis among adults with colon cancer in England. *JAMA Netw Open*. 2022;5(10):e2238569. doi:10.1001/jamanetworkopen.2022.38569

eTable 1. Symptom Code List
eAppendix. Routes to Diagnosis for Included Patients Who Were Symptomatic
eFigure. Study Variables and Hypothesized Associations Between Mental Health Morbidity and Symptomatic Presentations and Route to Cancer Diagnosis
eTable 2. Characteristics of 2115 Patients With Colon Cancer and Red-Flag Symptoms by Mental Health Morbidity (MHM) Status
eTable 3. Patients at Risk for Diagnosis and Investigation by Mental Health Morbidity (MHM) per Time Point Before Cancer Diagnosis
eTable 4. Likelihood of Being Diagnosed After Emergency Presentation (EP) in 2115 Patients With Red-Flag Symptoms
eTable 5. Likelihood of Being Diagnosed After Expedited Referral in Patients Aged 60 y and Older With Red-Flag Symptoms
eTable 6. Likelihood of Being Diagnosed After Emergency Presentation Among Patients With Colon Cancer Who Were Symptomatic

This supplemental material has been provided by the authors to give readers additional information about their work.

© 2022 Benitez Majano S et al. *JAMA Network Open.*

**eTable 1.** Symptom Code List		
**Symptom category**	**Medcode**	**Readcodes**
abdominal pain	11647	25C8.00
abdominal pain	3338	R090z00
abdominal pain	6395	196..12
abdominal pain	6357	197..14
abdominal pain	8436	R090H00
abdominal pain	2982	1978
abdominal pain	4617	1971
abdominal pain	7300	1979
abdominal pain	8362	R090K00
abdominal pain	5960	197..13
abdominal pain	17223	25C..12
abdominal pain	19283	R090N00
abdominal pain	22608	197C.00
abdominal pain	701	1975
abdominal pain	1976	196..11
abdominal pain	7490	197..11
abdominal pain	5782	25C..15
abdominal pain	716	R090400
abdominal pain	1228	1829
abdominal pain	36558	25C5.00
abdominal pain	25630	25C7.00
abdominal pain	628	R090700
abdominal pain	19020	25C..13
abdominal pain	29352	1969000
abdominal pain	1239	R090200
abdominal pain	4771	R090600
abdominal pain	177	1969
abdominal pain	17324	1DC5.00
abdominal pain	16868	R090A00
abdominal pain	37101	25C4.00
abdominal pain	15288	R090C00
abdominal pain	19360	R090M00
abdominal pain	16402	R090000
abdominal pain	16806	R090900
abdominal pain	7726	R090J00
abdominal pain	25118	197..00
abdominal pain	5691	1963
abdominal pain	34301	25D..00
abdominal pain	12639	25C2.00
abdominal pain	21583	25CA.00
abdominal pain	3086	1976
abdominal pain	24584	25D..11
abdominal pain	948	R090F00
abdominal pain	24661	197A.00
abdominal pain	23872	1973
abdominal pain	5804	2I18.12
abdominal pain	5899	1A53.12
abdominal pain	15213	2I18.00
abdominal pain	15180	25C..00
abdominal pain	20475	R090800
abdominal pain	11070	197A.11
abdominal pain	14916	25CZ.00
abdominal pain	290	1972
abdominal pain	3978	197B.00
abdominal pain	19223	25C3.00
abdominal pain	7812	1962
abdominal pain	2234	R090E00

© 2022 Benitez Majano S et al. *JAMA Network Open.*

abdominal pain	421	197..12
abdominal pain	1181	1977
abdominal pain	13626	2I18100
abdominal pain	542	R090500
abdominal pain	9695	197D.00
abdominal pain	17636	25C..14
abdominal pain	20640	25C..11
abdominal pain	2383	1968
abdominal pain	14989	196..00
abdominal pain	42211	25C9.00
abdominal pain	1763	R090.00
abdominal pain	2056	R090100
abdominal pain	3049	J574700
abdominal pain	1282	A081000
abdominal pain	7248	R090G12
abdominal pain	6717	14C4.00
abdominal pain	2767	J574800
abdominal pain	1561	J4...11
abdominal pain	20391	12E2.00
abdominal pain	23950	J41..11
bloating	29567	19A2.00
bloating	5150	R073400
bloating	14880	R073300
bloating	6229	19A..00
bloating	2657	19A3.00
bloating	5821	19B..12
bloating	41607	19AZ.00
change in bowel habit	16665	R078.00
change in bowel habit	19690	19EA.11
change in bowel habit	910	19EA.00
constipation	5803	J520z00
constipation	23641	J520000
constipation	1709	J520.00
constipation	6364	J520100
constipation	24180	J520y00
constipation	1028	19C..00
constipation	99999	J520400
constipation	25797	J520200
constipation	26022	J520300
constipation	17652	19C2.00
constipation	10687	J503100
constipation	2004	19C..11
constipation	20450	19CZ.00
diarrhoea	17017	J4zz.11
diarrhoea	14695	19FZ.00
diarrhoea	48313	A082z00
diarrhoea	7644	19G..00
diarrhoea	14881	R077100
diarrhoea	6685	J43z.11
diarrhoea	5090	A083.00
diarrhoea	6016	J4...13
diarrhoea	10158	E264311
diarrhoea	192	19F..11
diarrhoea	2133	A082000
diarrhoea	53739	A074011
diarrhoea	5036	J525.00
diarrhoea	11155	A082.11
diarrhoea	4343	19F2.00
diarrhoea	1695	19F..12
diarrhoea	15371	E264300

© 2022 Benitez Majano S et al. *JAMA Network Open.*

diarrhoea	14665	A083.11
diarrhoea	5134	19F..00
diarrhoea	29835	J521000
diarrhoea	2182	19FZ.11
diarrhoea	4542	A082.00
diarrhoea	52750	Ayu0H00
diarrhoea	30321	J4z..11
diarrhoea	21294	19F3.00
diarrhoea	8765	19F1.00
diarrhoea	15289	A076.11
fatigue	5583	R007000
fatigue	9656	R007211
fatigue	1688	R2y3.00
fatigue	6190	168..11
fatigue	15516	Eu46000
fatigue	16479	1684
fatigue	1147	R007500
fatigue	2855	E205.00
fatigue	6242	E205.12
fatigue	9889	1683.11
fatigue	5794	R007z11
fatigue	27877	R007.00
fatigue	7235	F286.11
fatigue	1371	R007300
fatigue	5751	168..00
fatigue	6029	F286.12
fatigue	7529	F286.14
fatigue	1582	R007100
fatigue	3361	F286.00
fatigue	1404	1682
fatigue	12411	2832.12
fatigue	1900	1B32.00
fatigue	9127	1688
fatigue	9220	Eu46011
fatigue	9435	1684.11
fatigue	4546	R007200
fatigue	1042	R007400
fatigue	44215	F286000
fatigue	17736	R204.00
fatigue	5814	1B3..12
fatigue	5049	168..12
fatigue	23932	F286.13
fatigue	29292	F286100
fatigue	5658	1683
fatigue	9823	R007411
jaundice	5996	2274.11
jaundice	25418	1675
jaundice	6000	1675.11
jaundice	29488	2274
jaundice	355	R024.00
jaundice	3121	J66y600
jaundice	18574	R024100
jaundice	2612	R024111
jaundice	18019	1675.12
lump/mass abd	34238	7H2C500
lump/mass abd	20387	25L..00
lump/mass abd	21575	25J8.00
lump/mass abd	25588	25J1.00
lump/mass abd	21301	25K..00
lump/mass abd	3015	R093100

© 2022 Benitez Majano S et al. *JAMA Network Open.*

lump/mass abd	1987	25Q3.00
lump/mass abd	7073	R093200
lump/mass abd	56675	25JZ.00
lump/mass abd	20827	25J7.00
lump/mass abd	16370	R093z00
lump/mass abd	8731	25J..00
lump/mass abd	64153	25R3.00
lump/mass abd	4800	R093000
lump/mass abd	17246	25K4.00
lump/mass abd	5838	R093.00
obstruction	18789	J50zz11
obstruction	28322	J50z400
obstruction	1221	J50zz15
obstruction	21648	J50zz14
obstruction	935	J50z.00
obstruction	1544	J50zz00
obstruction	1307	J50z500
obstruction	29118	J50y000
obstruction	1347	J50zz13
obstruction	33759	J50yz00
obstruction	21568	J50zz12
obstruction	20471	J50..00
obstruction	42547	J50y.00
obstruction	23478	SP14400
obstruction	18240	J50y.11
rectal bleeding	6151	19E6.11
rectal bleeding	9968	19ED.00
rectal bleeding	32446	J573100
rectal bleeding	12471	J68z.00
rectal bleeding	1642	J68z.11
rectal bleeding	6554	J573012
rectal bleeding	5462	19E6.00
rectal bleeding	6574	J573000
rectal bleeding	20859	J681.13
rectal bleeding	4636	J68zz00
rectal bleeding	621	J573011
rectal bleeding	45911	19EG.00
rectal bleeding	104124	8HTE000
rectal bleeding	19271	J573.00
rectal bleeding	11698	196C.00
rectal bleeding	3053	4794
rectal bleeding	2832	G848000
rectal bleeding	27862	J681.12
rectal bleeding	3872	J573.11
rectal bleeding	2873	J681.11
rectal bleeding	11718	196B.00
rectal bleeding	3097	J68..00
tenesmus	38499	19D2.00
tenesmus	15756	R07z000
tenesmus	6541	19D..11
tenesmus	14948	19D..00
weight loss	654	1623
weight loss	12398	1D1A.00
weight loss	102563	1627
weight loss	37937	22A8.00
weight loss	5812	1625.11
weight loss	24068	R2y4.00
weight loss	4663	1625
weight loss	126	22A6.00
weight loss	12530	R034800

© 2022 Benitez Majano S et al. *JAMA Network Open.*

weight loss	3647	R032.00

© 2022 Benitez Majano S et al. *JAMA Network Open.*

eAppendix. Routes to Diagnosis for Included Patients Who Were Symptomatic Based on NCIN-PHE)[22]

Fast-track (or two-week wait) referrals: Patients referred urgently by their GP for suspected cancer, so that they can see a specialist within 2 weeks (introduced in England in 2000).

Routine GP referrals: Patients referred by their GP but not under the two-week wait referral route

Elective outpatient/inpatient: An elective route starting with an outpatient appointment, either consultant to consultant referral or other referral; or starting with inpatient admission where no earlier information is available from waiting list prior to admission.

Emergency cancer diagnosis: Diagnosis of cancer following presentation to an Accident and Emergency Unit or a GP emergency referral or emergency pathways for in/out-patients.

© 2022 Benitez Majano S et al. *JAMA Network Open.*

eFigure. Study Variables and Hypothesized Associations Between Mental Health Morbidity and Symptomatic Presentations and Route to Cancer Diagnosis

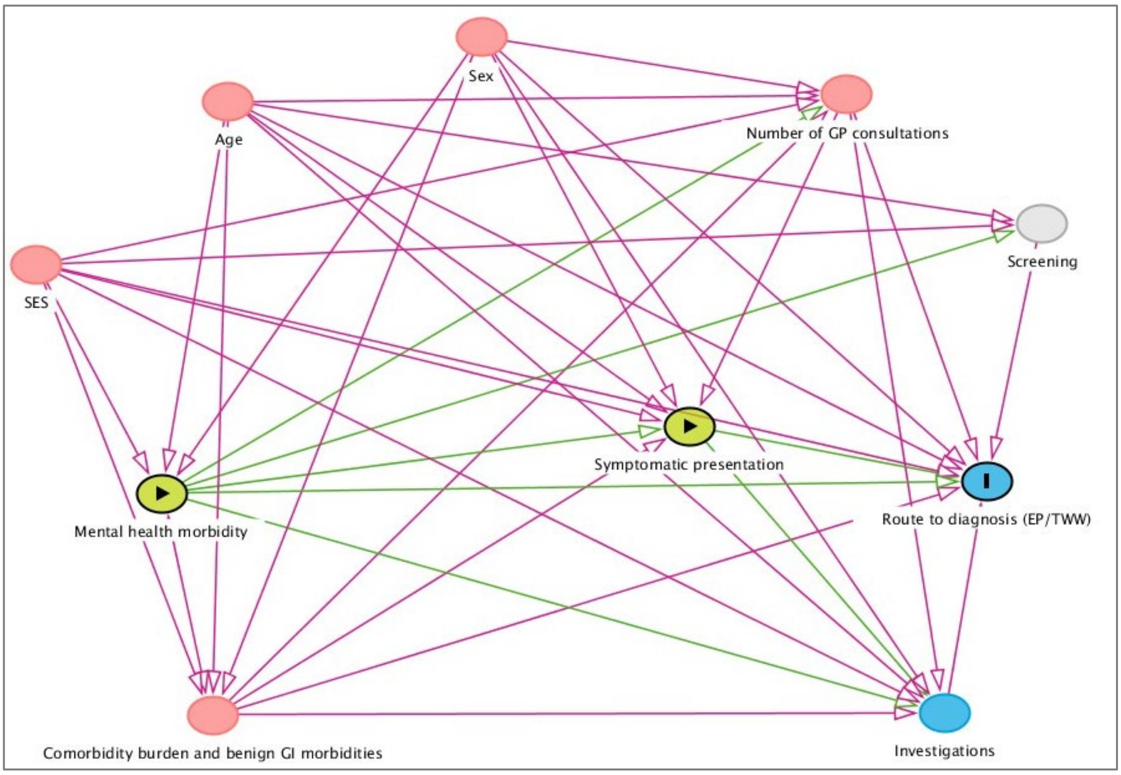

Notes: Investigations are considered as 'ancestor outcomes' (defined as an outcome that is 'upstream' and can be on the causal pathway with respect to the main outcome of interest); potential confounding variables include comorbidity burden, socio-economic status (SES), age, sex, and number of GP consultations for any reason as 'ancestors' of exposures and outcomes. Screening is considered as an 'unobserved ancestor', as the study is focusing on cancer diagnosis in symptomatic patients, rather than screening in non-symptomatic individuals.
Purple: biasing paths; Green: causal paths. (Produced with DAGitty v3.0)

eTable 2. Characteristics of 2115 Patients With Colon Cancer and Red-Flag Symptoms or Signs by Mental Health Morbidity (MHM) Status

	All patients N (%) (n = 2115)	No MHM N (%) (n = 1807)	MHM N (%) (n =308)	Chi2 p-value
**Age group**				0.097
<45	37 (1.7)	33 (1.8)	4 (1.3)	
45-54	133 (6.3)	113 (6.3)	20 (6.5)	
55-64	299 (14.1)	270 (14.9)	29 (9.4)	
65-74	520 (24.6)	447 (24.7)	73 (23.7)	
75-84	790 (37.4)	667 (36.9)	123 (39.9)	
85+	336 (15.9)	277 (15.3)	59 (19.2)	
**Sex**				0.000
Male	1,061 (50.2)	941 (52.1)	120 (39.0)	
Female	1,054 (49.8)	866 (47.9)	188 (61.0)	
**Deprivation quintile**				0.000
1 (Least deprived)	550 (26.0)	457 (25.3)	93 (30.2)	
2	483 (22.8)	434 (24.0)	49 (15.9)	
3	463 (21.9)	407 (22.5)	56 (18.2)	
4	366 (17.3)	309 (17.1)	57 (18.5)	
5 (most deprived)	253 (12.0)	200 (11.1)	53 (17.2)	
**Charlson Comorbidity Index**				0.000
0	1,155 (54.6)	1,035 (57.3)	120 (39.0)	
1	470 (22.2)	388 (21.5)	82 (26.6)	
2	247 (11.7)	194 (10.7)	53 (17.2)	
3+	243 (11.5)	190 (10.5)	53 (17.2)	
**Irritable Bowel Syndrome / Diverticular Disease**				0.162
No	1,926 (91.1)	1,652 (91.4)	274 (89.0)	
Yes	189 (8.9)	155 (8.6)	34 (11.0)	
**Visits to GP in 1-12 months prior cancer diagnosis**				0.000
0	27 (1.3)	25 (1.4)	2 (0.6)	
1-4	179 (8.5)	174 (9.6)	5 (1.6)	
5-9	367 (17.4)	342 (18.9)	25 (8.1)	
10+	1,542 (72.9)	1,266 (70.1)	276 (89.6)	
**Type of symptoms in 24 months prior cancer diagnosis**				0.434
Rectal bleeding or CIBH	860 (40.7)	741 (41.0)	119 (38.6)	
Anaemia (only red-flag)	1,255 (59.3)	1,066 (59.0)	189 (61.4)	
**Bowel endoscopy in 24 months prior cancer diagnosis**				0.083
No	508 (24.0)	422 (23.4)	86 (27.9)	
Yes	1,607 (76.0)	1,385 (76.6)	222 (72.1)	
**Route to diagnosis**				0.000
Emergency presentation	417 (19.7)	327 (18.1)	90 (29.2)	
Two-week wait	859 (40.6)	765 (42.3)	94 (30.5)	
GP referral	541 (25.6)	450 (24.9)	91 (29.5)	
Screening	63 (3.0)	63 (3.5)	0 (0.0)	
Inpatient elective	76 (3.6)	68 (3.8)	8 (2.6)	
Other outpatient	159 (7.5)	134 (7.4)	25 (8.1)	

© 2022 Benitez Majano S et al. *JAMA Network Open.*

eTable 3. Patients at Risk for Diagnosis and Investigation by Mental Health Morbidity (MHM) per Time Point Before Cancer Diagnosis

These numbers at risk refer to patients included in each panel in Figure 2.

	**All symptomatic patients**					**Patients with red-flag symptoms**			
	**No MHM**		**MHM**			**No MHM**		**MHM**	
	At Risk	Diagnosed	At Risk	Diagnosed		At Risk	Diagnosed	At Risk	Diagnosed
Baseline	3143		623	0		1807	0	308	0
180 days	1583	1560	401	222		814	993	186	122
365 days	1080	503	301	100		519	295	139	47
545 days	637	443	186	115		282	237	80	59
730 days	0	637	0	186		0	282	0	80
	At Risk	Investigated	At Risk	Investigated		At Risk	Investigated	At Risk	Investigated
Baseline	2336	0	439	0		1485	0	245	0
180 days	937	1399	217	222		502	983	111	134
365 days	595	342	146	71		299	203	76	35
545 days	310	285	74	72		149	150	41	35
730 days	0	310	0	74		0	149	0	41

© 2022 Benitez Majano S et al. *JAMA Network Open.*

**eTable 4.** Likelihood of Being Diagnosed After Emergency Presentation (EP) in 2115 Patients With Red-Flag Symptoms					
	**% diagnosed following EP**	**Unadjusted OR**		**Adjusted OR**	
		OR	95% CI	OR	95% CI
**Mental Health Morbidity**					
No	327 (18.1)				
Yes	90 (29.2)	1.9	1.4,2.4	1.6	1.2,2.2
**Age group**					
<45	5 (13.5)	0.9	0.3,2.2	0.9	0.3,2.4
45-54	21 (15.8)	1.1	0.6,1.8	1.0	0.6,1.7
55-64	45 (15.1)				
65-74	69 (13.3)	0.9	0.6,1.3	0.7	0.5,1.1
75-84	158 (20.0)	1.4	1.0,2.0	1.0	0.7,1.5
85+	119 (35.4)	3.1	2.2,4.4	2.2	1.5,3.3
**Sex**					
Male	186 (17.5)				
Female	231 (21.9)	1.1	0.6,1.8	1.0	0.6,1.7
**Deprivation quintile**					
1	94 (17.1)				
2	95 (19.7)	1.2	0.9,1.6	1.3	0.9,1.8
3	81 (17.5)	1.0	0.8,1.4	1.1	0.8,1.5
4	76 (20.8)	1.3	0.9,1.8	1.3	0.9,1.9
5	71 (28.1)	1.9	1.3,2.7	1.8	1.3,2.7
**CCI score**					
0	148 (12.8)				
1	117 (24.9)	2.3	1.7,2.9	2.1	1.6,2.8
2	53 (21.5)	1.9	1.3,2.6	1.6	1.1,2.4
3+	99 (40.7)	4.7	3.4,6.4	4.0	2.8,5.6
**Irritable Bowel Syndrome / Diverticular Disease**					
No	371 (19.3)				
Yes	46 (24.3)	1.3	1.0,1.9	1.3	0.9,1.8
**Visits to GP in 1-12 months prior cancer diagnosis**					
0	8 (29.6)	1.6	0.7,3.6	1.8	0.7,4.7
1-4	34 (19.0)	0.9	0.6,1.3	1.9	1.2,3.2
5-9	49 (13.4)	0.6	0.4,0.8	1.0	0.7,1.4
10+	326 (21.1)				
**Type of symptoms in 24 months prior cancer diagnosis**					
Rectal bleeding or CIBH	118 (13.7)				
Anaemia (only red-flag)	299 (23.8)	2.0	1.6,2.5	1.9	1.5,2.4

Logistic regression Odds Ratios (OR) and 95% Confindence Intervals (95%CI)

© 2022 Benitez Majano S et al. *JAMA Network Open.*

**eTable 5.** Likelihood of Being Diagnosed After Expedited Referral in Patients Aged 60 y and Older With Red-Flag Symptoms					
	**% diagnosed following TWW**	**Unadjusted OR**		**Adjusted OR**	
		OR	95% CI	OR	95% CI
**Mental Health Morbidity**					
No	650 (42.4)				
Yes	76 (27.9)	0.5	0.4,0.7	0.6	0.5,0.8
**Age group**					
55-64	72 (45.3)				
65-74	217 (41.7)	0.9	0.6,1.3	1.0	0.7,1.6
75-84	333 (42.2)	0.9	0.6,1.3	1.2	0.8,1.8
85+	104 (31.0)	0.5	0.4,0.8	0.8	0.5,1.2
**Sex**					
Male	368 (41.2)				
Female	358 (39.3)	0.9	0.8,1.1	1.0	0.8,1.2
**Deprivation quintile**					
1	193 (41.5)				
2	162 (38.8)	0.9	0.7,1.2	0.8	0.6,1.1
3	190 (48.6)	1.3	1.0,1.7	1.3	1.0,1.7
4	111 (35.5)	0.8	0.6,1.0	0.8	0.6,1.0
5	70 (32.0)	0.7	0.5,0.9	0.7	0.5,1.0
**CCI score**					
0	423 (46.1)				
1	168 (40.1)	0.8	0.6,1.0	0.9	0.7,1.2
2	81 (34.2)	0.6	0.4,0.8	0.8	0.5,1.1
3+	54 (23.3)	0.4	0.3,0.5	0.5	0.3,0.7
**Irritable Bowel Syndrome / Diverticular Disease**					
No	683 (41.7)				
Yes	43 (25.4)	0.5	0.3,0.7	0.5	0.3,0.7
**Visits to GP in 1-12 months prior cancer diagnosis**					
0	6 (30.0)	0.8	0.3,2.0	0.8	0.3,2.2
1-4	68 (57.6)	2.4	1.6,3.5	1.8	1.2,2.7
5-9	149 (53.6)	2.0	1.6,2.6	1.6	1.2,2.1
10+	503 (36.2)				
**Type of symptoms in 24 months prior cancer diagnosis**					
Rectal bleeding or CIBH	310 (43.9)				
Anaemia (only red-flag)	416 (37.9)	0.8	0.6,1.0	0.8	0.7,1.0

Logistic regression Odds Ratios (OR) and 95% Confindence Intervals (95%CI)

eTable 6. Likelihood of Being Diagnosed After Emergency Presentation Among Patients With Colon Cancer Who Were Symptomatic

	Emergency diagnosis n (%)	Unadjusted OR		Adjusted OR	
		OR	95% CI	OR	95% CI
**Mental Health Morbidity**					
No	859 (27.3)				
Yes	231 (37.1)	1.6	1.3,1.9	1.4	1.2,1.7
**Age group**					
<45	30 (35.3)	1.7	1.1,2.6	1.5	0.9,2.5
45-54	71 (28.0)	1.2	0.9,1.6	1.1	0.8,1.5
55-64	133 (24.7)				
65-74	220 (23.4)	0.9	0.7,1.2	0.9	0.7,1.2
75-84	369 (28.0)	1.2	0.9,1.5	1.2	0.9,1.5
85+	267 (42.4)	2.2	1.8,2.9	2.1	1.6,2.8
**Sex**					
Male	501 (27.0)				
Female	589 (30.8)	1.2	0.9,1.6	1.1	0.8,1.5
**Deprivation quintile**					
1	260 (27.5)				
2	250 (28.7)	1.1	0.9,1.3	1.1	0.9,1.3
3	210 (26.1)	0.9	0.8,1.1	1.0	0.8,1.2
4	193 (29.4)	1.1	0.9,1.3	1.0	0.8,1.3
5	177 (36.3)	1.5	1.2,1.9	1.4	1.1,1.8
**CCI score**					
0	429 (21.9)				
1	295 (33.7)	1.8	1.5,2.2	2.0	1.6,2.4
2	127 (30.4)	1.6	1.2,1.9	1.8	1.4,2.3
3+	239 (46.3)	3.1	2.5,3.8	3.4	2.6,4.3
**Irritable Bowel Syndrome / Diverticular Disease**					
No	983 (28.6)				
Yes	107 (32.0)	1.2	0.9,1.5	1.2	0.9,1.5
**Visits to GP in 1-12 months pre-cancer diagnosis**					
0	35 (39.3)	1.7	1.1,2.6	1.5	0.9,2.5
1-4	119 (36.3)	1.5	1.1,1.9	2.5	1.9,3.4
5-9	186 (28.1)	1.0	0.8,1.2	1.5	1.2,1.8
10+	750 (27.9)				
**Symptoms in 24 months pre-cancer diagnosis**					
Rectal bleeding or CIBH	118 (13.7)				
Anaemia (only red-flag)	299 (23.8)	2.0	1.6,2.5	1.9	1.5,2.4
Non red-flag only	538 (44.1)	5.0	4.0,6.2	5.0	4.0,6.3
Chronic only	135 (31.3)	2.9	2.2,3.8	2.1	1.6,2.8

© 2022 Benitez Majano S et al. *JAMA Network Open.*
